# Sequence-Specific Fidelity Alterations Associated with West Nile Virus Attenuation in Mosquitoes

**DOI:** 10.1371/journal.ppat.1005009

**Published:** 2015-06-26

**Authors:** Greta A. Van Slyke, Jamie J. Arnold, Alex J. Lugo, Sara B. Griesemer, Ibrahim M. Moustafa, Laura D. Kramer, Craig E. Cameron, Alexander T. Ciota

**Affiliations:** 1 The Arbovirus Laboratory, Wadsworth Center, New York State Department of Health, Slingerlands, New York, United States of America; 2 Department of Biochemistry and Molecular Biology, The Pennsylvania State University, University Park, Pennsylvania, United States of America; 3 Department of Biomedical Sciences, State University of New York at Albany School of Public Health, Albany, New York, United States of America; University of Texas Medical Branch, UNITED STATES

## Abstract

High rates of error-prone replication result in the rapid accumulation of genetic diversity of RNA viruses. Recent studies suggest that mutation rates are selected for optimal viral fitness and that modest variations in replicase fidelity may be associated with viral attenuation. Arthropod-borne viruses (arboviruses) are unique in their requirement for host cycling and may necessitate substantial genetic and phenotypic plasticity. In order to more thoroughly investigate the correlates, mechanisms and consequences of arbovirus fidelity, we selected fidelity variants of West Nile virus (WNV; *Flaviviridae*, *Flavivirus*) utilizing selection in the presence of a mutagen. We identified two mutations in the WNV RNA-dependent RNA polymerase associated with increased fidelity, V793I and G806R, and a single mutation in the WNV methyltransferase, T248I, associated with decreased fidelity. Both deep-sequencing and *in vitro* biochemical assays confirmed strain-specific differences in both fidelity and mutational bias. WNV fidelity variants demonstrated host-specific alterations to replicative fitness *in vitro*, with modest attenuation in mosquito but not vertebrate cell culture. Experimental infections of colonized and field populations of *Cx*. *quinquefaciatus* demonstrated that WNV fidelity alterations are associated with a significantly impaired capacity to establish viable infections in mosquitoes. Taken together, these studies (i) demonstrate the importance of allosteric interactions in regulating mutation rates, (ii) establish that mutational spectra can be both sequence and strain-dependent, and (iii) display the profound phenotypic consequences associated with altered replication complex function of flaviviruses.

## Author Summary

West Nile virus (WNV) is the most geographically widespread arthropod-borne virus (arbovirus) in the world. Like most arboviruses, WNV is a RNA virus which is highly mutable and exists in nature as genetically diverse mutant swarms. Although many recent studies have investigated the relationship between virus mutation rate and viral fitness, this had not previously been determined for WNV or other flaviviruses. We identified WNV mutations associated with variation in mutation rate using cell culture passage in the presence of a mutagen and engineered these mutations into an infectious WNV clone in order to investigate the causes and consequences of altered fidelity. Our results demonstrate that interactions among proteins which comprise the WNV replication complex can significantly alter both the extent and types of mutations that occur. In addition, we show that both increasing and decreasing WNV fidelity has host-specific effects on replication in cell culture and is associated with nearly complete ablation of WNV infection in mosquito vectors. These results have significant implications for our understanding of arbovirus evolution, replication complex function and arboviral fitness in mosquitoes, and identify important targets to study the determinants and mechanisms of vector competence and arbovirus fidelity.

## Introduction

Lack of proofreading mechanisms and high replication rates among most RNA viruses make them inherently error-prone, yet there is also variation in mutation rates among both species and strains of RNA viruses, making fidelity itself a trait with a genetic basis subject to some fine-tuning by selection [1–3]. The generally accepted belief is that genetic diversity provides a benefit for RNA viruses for which success depends on the capacity to effectively proliferate in a range of internal environments and evade host immunity [4–6]. Such plasticity could be particularly beneficial for arthropod-borne viruses (arboviruses), which require successful infection, replication and transmission by taxonomically divergent vertebrate and invertebrate hosts.

On the other hand, some would argue that mutation rate is simply coupled to replication rate and that the low fidelity of RNA viruses is not a requirement, but rather a consequence of selection for maximum replicative fitness [7]. There is indeed a clear relationship between replication fidelity and replication rate [8], but there is also evidence that the two can be uncoupled. For example, the high-fidelity variant of poliovirus (PV), G64S, was shown to have replicative kinetics equivalent to wildtype virus *in vitro* [9–11]. Pushing mutation rate beyond maximum replicative fitness creates a scenario in which genetic information is lost and selection can no longer outpace the accumulation of deleterious mutations, termed lethal mutagenesis [12,13]. Selection for mutational robustness could buffer somewhat against the negative impacts of increased mutational load, yet there is clearly a limit to this, as demonstrated by the effectiveness of ribavirin and other mutagenic antivirals against a range of RNA viruses [14,15]. In addition, previous studies demonstrate that mutator variants of chikungunya virus (CHIKV), coxsackievirus (CV), SARS-coronavirus and PV are highly attenuated [16–19]. Conversely, high-fidelity variants of PV, CV, foot and mouth disease virus and CHIKV have also been shown to be attenuated in various hosts [10,20–25], suggesting that there is likely a delicate balance between the need for accuracy and diversity among RNA viruses.

With the exception of important studies with CHIKV, studies directly evaluating the phenotypic impact of mutation rates of arboviruses are lacking. Given the species-specific differences in selective pressure and virus-host interactions, there is clearly a need to individually characterize these relationships for other medically important arboviruses [26]. In addition, direct evidence linking specific arbovirus mutations to biochemical alterations affecting fidelity have not been presented, and therefore the mechanism of altered fidelity, including the role of specific structural changes in the RNA-dependent RNA polymerase (RdRp) and allosteric interactions with other proteins in the replication complex, are not well defined. Lastly, there is intriguing evidence that not just the effect of altered mutation rate, but fidelity itself could be host or even cell-specific [27], which could be particularly relevant for arboviruses.

West Nile virus (WNV; *Flaviviridae*, *Flavivirus*) is the most geographically widespread arbovirus in the world and there remains no effective therapeutics or prophylactics against WNV disease in humans. Although WNV is one of the most well characterized arboviruses in terms of evolution and host-virus interactions, there are gaps in our understanding of host-specific selective pressures and genetic correlates of viral fitness and pathogenesis. While there is evidence of superior WNV fitness in mosquitoes with highly homogeneous strains [28], and the accumulation of diversity in mosquitoes could simply be a product of relaxed purifying selection as a result of mutational robustness [29], there is also evidence for a correlation between WNV fitness and intrahost diversity in mosquito cell culture and *Culex* mosquitoes [30–32]. Increased diversity has also been associated with decreased WNV virulence in mice [31], suggesting that altering the capacity to accumulate mutations could have host-specific phenotypic consequences.

In order to gain insight into the phenotypic correlates and mechanism of WNV mutation restriction and expansion, we utilized experimental evolution in the presence of the antiviral ribavirin to identify mutations in the WNV replication complex important in regulating fidelity and characterized WNV mutants possessing these changes. Our results provide new insight into the specificity of genome replication and fidelity, the importance of allosteric changes in the regulation of mutation, and the host-specific consequences of alterations to fidelity.

## Materials and Methods

### Selection and resistance assays

A WNV infectious clone (WNV-IC), generated from WNV strain 3356 (NY99; AF404756) as previously described [33] was serially passaged in HeLa cells (ATCC) in the presence of the antiviral nucleoside analog ribavirin (Sigma) in duplicate (Lineage I & II). Both lineages were passaged in the presence of 50, 100 and 250uM ribavirin, and the virus with the highest infectious titer at 5 days post-infection (pi) was used to initiate the subsequent passage at all concentrations of ribavirin. Ribavirin-treated HeLa cell monolayers were also infected with fresh stock of WNV-IC at each passage as a naive control for comparative antiviral resistance of serially passaged virus. A multiplicity of infection (MOI) of 0.1 was used to initiate all passages and resistance assays. In addition to ribavirin, susceptibility to 50uM 5-fluorouracil (Sigma) was also determined for select WNV strains. HeLa cells were grown in EMEM supplemented with 100ug/ml penicillin streptomycin and 2% fetal bovine serum (FBS). For all cells treated with antiviral compounds growth media was removed and monolayers in 6-well cell culture plates were overlaid with 1ml media containing the antiviral compound and incubated for 1h at 37°C. Media was then removed and replaced with 100ul of virus diluted in media supplemented with antiviral compound and incubated for 1 hour at 37°C. After incubation, 3mls of media supplemented with desired concentration of antiviral was added to each well. Supernatants were harvested at day 5 pi and titrated by plaque assay on African Green Monkey kidney (Vero) cells (ATCC) according to standard protocol [34]. In order to isolate clonal strains with decreased antiviral susceptibility, 20 individual plaques were harvested from Vero monolayers following the completion of passage 6, re-suspended in 100ul of EMEM, inoculated onto fresh Vero monolayers and grown in liquid culture for 96h. Susceptibility of mutagens was reported as log_10_ reduction and titer and compared using t-tests following confirmation of normality (GraphPad Prism, Version 5.0).

### Sequencing

Full-genome consensus sequencing was performed in order to determine changes accrued with passage and to verify sequences of mutated viruses. RNA was extracted from cell culture supernatant and subjected to reverse transcription (RT) and polymerase chain reactions (PCR) using the SuperScript III one-step RT-PCR kit (Life technologies) with 5–10 overlapping fragments (sequences available upon request). RT-PCR products were concentrated using Zymo-5 DNA spin columns (Zymo Research). Sequencing was carried out using the same RT-PCR primer sets and all sequencing reactions were completed at the Wadsworth Center Applied Genomics Technology Core (WCAGTC) on an ABI 3100 or 3700 automated sequencer (Applied Biosystems). WNV amplicons of nucleotides 1311–3248 (envelope/ns1 genes; [29]) were created for deep-sequencing using the same methodology with WNV RNA isolated following 72 h growth on *Aedes albopictus* cells (C6/36, ATCC). C6/36 cells were used in order to maximize viral titer for sequencing and were grown in MEM supplemented with 10% FBS, 2 mM L-glutamine, 1.5 g/L sodium bicarbonate, 0.1mM non-essential amino acids, 100 U/ml of penicillin, and 100 ug/ml of streptomycin and maintained at 28°C in 5% CO_2_. Deep-sequencing was performed at the WCAGTC on the Ion Torrent Personal Genome Machine (IT-PGM) using a 316 semiconductor chip.

Sequences were compiled and edited using the SeqMan module of the DNAStar software package (DNAStar) and a minimum of two-fold redundancy throughout each clone or consensus fragment was required for sequence data to be considered complete. Ion Torrent generated sequence data was analyzed by the Wadsworth Center Bioinformatics Core facility using CLC Genomics Workbench (CLC bio) software. Quality trimming of sequence reads, reference mapping and SNP (single-nucleotide polymorphism) detection was done in CLC Genomics Workbench v5.0.1. Quality trimming and reference mapping were done with default parameters. Reference mapping was completed using the WNV-IC sequence (AF404756). SNP detection was performed with default parameters except minimum coverage was set to 20, minimum variant frequency was set to 1.0% and ploidy was set to 1.

### Site-directed mutagenesis

WNV mutants including C8423T (T248I), G10057A (V793I), and G10096A (G806R) were generated by site-directed mutagenesis (SDM) of the WNV-IC using mutagenic primer sets along with the QuikChange XLII SDM kit (Stratagene) as per the manufacturer’s protocol. Mutant WNV-IC DNA was then amplified in *E*. *coli* and plasmid harvested by Highspeed Midiprep (Qiagen). Full-genome sequencing of NS5 mutant WNV-IC plasmids indicated no other mutations were present except those engineered. Transcription of mutant and control WNV-IC plasmids was carried out by linearization with Xba1 and transcription using the MEGAscript kit (Ambion) supplemented with Anti-reverse cap analog (Ambion) and assembled as per manufacturer’s protocol. Transcription reactions were incubated at 37°C for 4h. Resulting RNA was purified with the MEGAclear kit (Ambion) and quantified on a Nanodrop 2000 (Thermo Fisher Scientific). RNA was stored in 10μg aliquots at -80°C. Wild-type WNV-IC RNA and mutant RNA were electroporated into 0.8 x 10^7^ C6/36 cells in PBS using a GenePulser (BioRad). Transfected cells were seeded into T75 flasks and supernatants were collected from day 3 to 7 post-transfection, aliquoted and stored at -80°C. WNV infectious particles were quantified by plaque titration on Vero cells.

### Construction of WNV NS5 bacterial expression plasmids

The WNV NS5 gene was cloned into the pET26Ub-CHIS bacterial expression plasmid [35]. This system allows for the production of ubiquitin fusion proteins containing a carboxy-terminal hexahistidine tag that are then co and/or post-translationally processed by the ubiquitin protease, co-expressed from a second plasmid, pUBPS. Briefly, the WNV NS5 coding region was amplified using the WNV NY99 strain (AF404756) as template, oligonucleotides 1 and 2 (Table 1) and Deep Vent DNA polymerase (NEB). The PCR product of WNV NS5 was gel purified and cloned into the pET26Ub-CHIS plasmid using SacII and BamHI sites and by using In-Fusion ligation independent cloning. The final construct (pET26Ub-WNV NS5-CHIS) was confirmed by sequencing at the Pennsylvania State University’s Nucleic Acid Facility. Expression plasmids for the WNV NS5 derivatives (T248I and V793I, G806R) were constructed using the same strategy.

**Table 1:** DNA oligonucleotides used for cloning WNV NS5 into bacterial expression plasmid.

	DNA Oligonucleotide	Sequence
1	Ub-SacII-INF-WNV-NS5-for	5’TG GTC CTG CGT CTC CGC GGT GGA GGT GGG GCA AAA GGA CGC3’
2	WNV-NS5-INF-BamHI-CHIS-rev	5’G GTG ACC AGA GGA TCC CAG TAC TGT GTC CTC AAC-3’

### Expression and purification of WNV NS5

E. coli Rosetta(DE3)pUBPS cells were transformed with the pET26Ub-WNV-NS5-CHIS plasmid for protein expression. Rosetta(DE3)pUBPS cells containing the pET26Ub-WNV-NS5-CHIS plasmid were grown in 100 mL of media (NZCYM) supplemented with kanamycin (25 μg/mL), chloramphenicol (20 μg/mL) and spectinomycin (S50) at 37°C until an OD_600_ of 1.0 was reached. This culture was then used to inoculate 1L of K75, C60, S150-supplemented ZYP-5052 auto-induction media studier [36], to an OD_600_ = 0.1. The cells were grown at 37°C to an OD_600_ of 0.8 to 1.0, cooled to 15°C and then grown for 36–40 h. Typically, after 36–40 h at 15°C the OD_600_ reached ~7.0–10.0. Cells were harvested by centrifugation (6000 x *g*, 10 min) and the cell pellet was washed once in 200 mL of TE (10 mM Tris, 1 mM EDTA), centrifuged again, and the cell paste weighed. Typically, yields were 20 g of cell paste per liter of culture. The cells were then frozen and stored at -80°C until used. Frozen cell pellets were thawed on ice and suspended in lysis buffer (100 mM potassium phosphate, pH 8.0, 500 mM NaCl, 5 mM 2-mercaptoethanol, 20% glycerol, 1.4 μg/mL leupeptin, 1.0 μg/mL pepstatin A and one Roche EDTA-free protease tablet per 10 g cell pellet), with 5 mL of lysis buffer per gram of cells. The cell suspension was lysed by passing through a French press (SLM-AMINCO) at 15,000 psi. After lysis, phenylmethylsulfonylfluoride (PMSF) and NP-40 were added to a final concentration of 1 mM and 0.1% (v/v), respectively. While stirring the lysate, polyethylenimine (PEI) was slowly added to a final concentration of 0.25% (v/v). The lysate was stirred for an additional 30 min at 4°C after the last addition of PEI, and then centrifuged at 75,000 x g for 30 min at 4°C. The PEI supernatant was decanted to a fresh beaker, and while stirring, pulverized ammonium sulfate was slowly added to 60% (w/v) saturation. This supernatant was stirred for 30 min after the last addition of ammonium sulfate, and centrifuged at 75,000 x g for 30 min at 4°C. The supernatant was decanted, and the pellet was suspended in buffer A (100 mM potassium phosphate, pH 8.0, 500 mM NaCl, 5 mM 2-mercaptoethanol, 20% glycerol, 1.4 μg/mL leupeptin, 1.0 μg/mL pepstatin A, 5 mM imidazole). The resuspended ammonium sulfate pellet was loaded onto a Ni-NTA column (Qiagen) at a flow rate of 1 mL/min (approximately 1 mL bed volume/100 mg total protein) equilibrated with buffer A. After loading, the column was washed with fifty column volumes of buffer A and five column volumes of buffer A containing 50 mM imidazole. Protein was eluted from the Ni-NTA column with buffer A containing 500 mM imidazole. Fractions were collected and assayed for purity by SDS-PAGE. Fractions with the highest purity were pooled and dialyzed against buffer B (50 mM HEPES pH 7.5, 500 mM NaCl, 5 mM 2-mercaptoethanol and 20% glycerol; MWCO of 24,000 Da). The dialyzed protein was then passed thru a Hi-Load 16/600 Superdex 200 prep grade gel filtration column (GE Healthcare) equilibrated with buffer B at 1 ml/min using an AktaPrime system. Fractions (3 mL) were collected, assayed for purity by SDS-PAGE, pooled and then concentrated to 150 μM (~15 mg/mL) using a Vivaspin concentrator (30,000 MWCO). The protein concentration was determined by measuring the absorbance at 280 nm by using a Nanodrop spectrophotometer and using a calculated molar extinction coefficient of 221,730 M^-1^ cm^-1^. Purified, concentrated protein was aliquoted and frozen at -80°C until use. Typical WNV NS5 yields were 1 mg/5 g of *E*. *coli* cells, which can be produced from 0.25 L of culture.

### WNV NS5 catalyzed nucleotide incorporation assays

To assemble WNV NS5 elongation competent complexes, 1 or 5 μM WNV NS5 was mixed with 10 μM pGGC RNA primer, 1 μM RNA template (either 5’-AAACUGAGAAGGAGAAAGCC-3’ or 5’-AAAUCGAGAAGGAGAAAGCC-3’), 20 μM CTP, 20 μM UTP and 0.1 μCi/μL [γ-^32^P]-UTP for 30 min in 50 mM HEPES pH 7.5, 5 mM MgCl_2_ and 10 mM 2-mercaptoethanol. For single nucleotide incorporation assays, the NS5 elongation competent complex was mixed with 25 μM heparin, 50 mM NaCl and 100 μM NTP substrate (either ATP or GTP) in 50 mM HEPES pH 7.5, 5 mM MgCl_2_ and 10 mM 2-mercaptoethanol at 30°C. After mixing, reactions were quenched at various times by the addition of 50 mM EDTA. Products were resolved from substrates by denaturing PAGE. An equal volume of loading buffer, 5 μL, (70% formamide, 0.025% bromophenol blue and 0.025% xylene cyanol) was added to 5 μL of quenched reaction mixtures and heated to 70°C for 2–5 min prior to loading 5 μL on a denaturing 20% polyacrylamide gel containing 1X TBE (89 mM Tris base, 89 mM boric acid, 2 mM EDTA) and 7 M Urea. Electrophoresis was performed in 1X TBE at 90 W. Gels were visualized by using a PhosphorImager (GE) and quantified by using ImageQuant software (GE).

Data were fit by nonlinear regression using the program KaleidaGraph (Synergy Software). All experiments shown are representative, single experiments that have been performed after at least three individual trials to define the concentration or time range shown. In all cases, values for parameters measured during individual trials were within the limits of the error reported for the final experiments. Kinetic data were fit by nonlinear regression using the program KaleidaGraph (Synergy Software, Reading, PA). Observed rate constants (*k*_obs_) for nucleotide incorporation were obtained by fitting product-versus-time data to an equation defining a single exponential (Eq 1), where A is the amplitude, *k*_obs_ is the observed rate constant and C is the endpoint.

Product=A*e (−kobs*t)+ C

### *In vitro* growth kinetics

Confluent monolayers of baby hamster kidney cells (BHK; ATCC) and *Culex tarsalis* mosquito cells (CxT; kindly provided by A. Brault, CDC Fort Collins) were infected with virus, in triplicate, using 6-well plates, at a MOI of 0.01 pfu/cell (multi-step), 10.0 pfu/cell (BHK one-step), or 8.0 pfu/cell (CxT one-step). BHK cells were grown in minimal essential medium (MEM, Gibco) supplemented with 10% fetal bovine serum, 2 mM L-glutamine, 1.5g/L sodium bicarbonate, 100 U/ml of penicillin, and 100 ug/ml of streptomycin and maintained at 37°C in 5% CO_2_. CxT cells were grown in Schneider’s media (GIBCO) supplemented with 10% FBS, 2 mM L-glutamine, 1.5 g/L sodium bicarbonate and maintained at 28°C in 5% CO_2_. After a one hour absorption period at 37°C (BHK) or 28°C (CxT), the inoculum was removed, cells were gently washed, overlaid with 2 ml of maintenance media and incubated at appropriate temperatures. Samples consisting of 50ul supernatant were harvested at 24, 48, 72, 96, and 120 (CxT only) hpi for multi-step growth kinetics and 3, 6, 9, 12, 24 and 30 (CxT only) hpi for one-step kinetics, diluted 1:10 in media containing 20% FBS, and stored at -80°C. Titrations were performed in duplicate, by plaque assay on Vero cells and mean titers for each time point were calculated. WNV RNA genomes were also quantified following extraction with QIAamp viral RNA spin columns (Qiagen) using a TaqMan quantitative real-time RT-PCR assay (Applied Biosystems) with a primer/probe designed for WNV E gene amplification [37]. Growth kinetics were compared using repeated measured ANOVA and Tukey’s post hoc tests and infectivity was compared by t-test following confirmation of normality (GraphPad Prism, Version 5.0).

### Invertebrate infection studies

Mosquito susceptibility was determined as previously described [38] in both highly colonized *Cx*. *quinquefasciatus* originally obtained from Benzon Research Inc. and F4 *Cx*. *quinquefasciatus* collected as egg rafts from Orange County, CA (kindly provided by Robert Cummings, Orange County California Vector Control District). Briefly, individual WNV strains were diluted to equivalent titers (~8.0 log_10_ pfu/ml), mixed 1:5, virus: defibrinated sheep blood (Colorado Serum) + 2.5% sucrose, and offered to ~500 female mosquitoes using a Hemotek membrane feeding system (Discovery Workshops). Following 1 h, mosquitoes were anesthetized using CO_2_ and fully engorged mosquitoes were saved and housed at 27°C for subsequent testing. Twenty-five to 50 mosquitoes per strain were saved at -80°C in 1 ml mosquito diluent [MD; 20% heat-inactivated fetal bovine serum (FBS) in Dulbecco’s phosphate-buffered saline (PBS) plus 50 μg/ml penicillin/streptomycin, 50 μg/ml gentamicin, and 2.5 μg/ml Fungizone] at days 5, 7, 10, 14 and 21 days post-feeding. Samples were thawed and homogenized for 30 seconds at 24hz in a Mixer Mill MM301 (Retsch). Debris was then pelleted by centrifugation at 6000 rcf for 5 minutes and the supernatant screened by plaque assay on Vero cells to determine infection status.

## Results

### WNV strain selection and mutagen resistance

**Fig 1:**
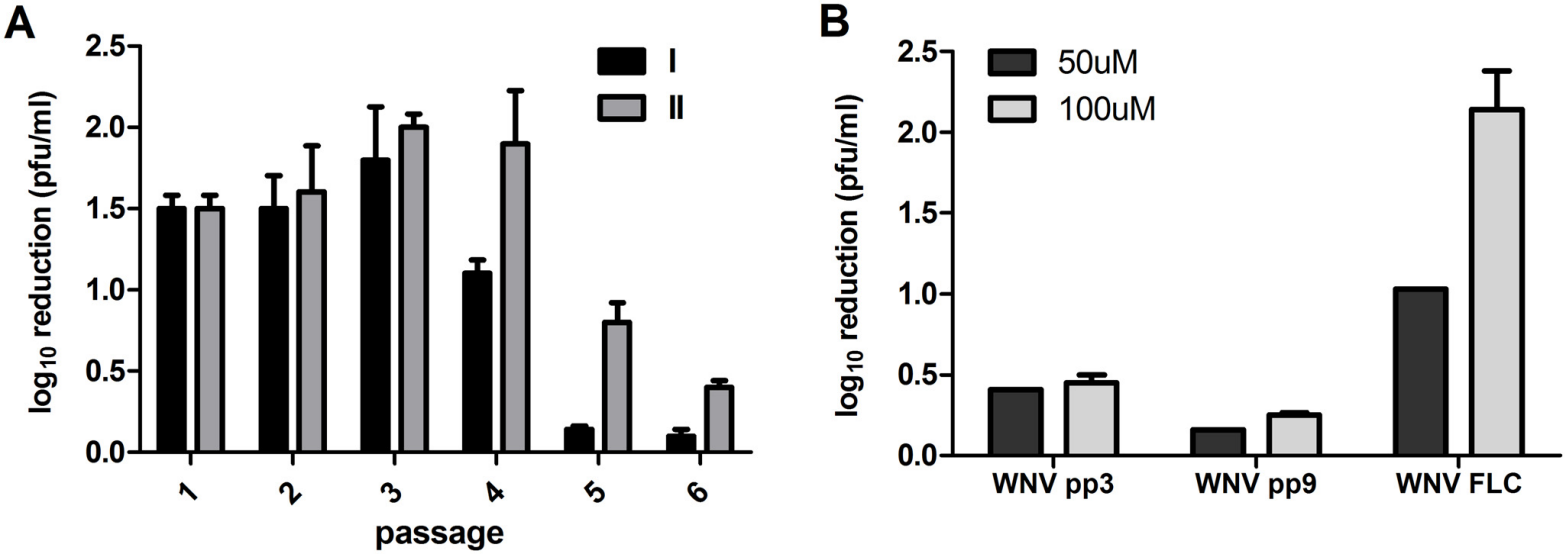
Selection for West Nile virus populations with decreased susceptibility to ribavirin. **A.** Ribavirin susceptibility, measured as mean log_10_ pfu/ml reduction in viral titer +/- SD resulting from 50uM ribavirin treatment for 72h on Hela cell culture relative to untreated controls, following passage of parallel series of ribavirin-treated WNV-IC designated as lineage I (black) and lineage II (grey). **B.** Ribavirin susceptibility in selected clonal populations. Clonal populations were created with lineage I from 20 plaques isolations (pp) and strains with the highest levels of resistance (WNV pp3/9) were chosen for further characterization.

Passaging in the presence of the antiviral ribavirin was used to select for WNV variants with decreased susceptibility and putative alterations to polymerase fidelity. Ribavirin susceptibility, as measured by reduction in viral titer following treatment, was monitored throughout the passage series and again assessed following the completion of passage 6 (Fig 1A). Significantly lower reductions in viral titer relative to WNV-IC controls were measured in both lineage I and II following passage 5 and 6 (t-test, df = 5, p<0.05), such that lineage I viral titer decreased just 1.4-fold following ribavirin treatment after 6 passages, as compared to a greater than 25-fold titer reduction measured for WNV-IC. In order to select for clonal strains with decreased mutagen susceptibility, ribavirin sensitivity was assessed for individual biological clones isolated from the lineage I passed virus and strains with the highest levels of resistance (WNV pp3, pp9; Fig 1B) were chosen for further characterization.

**Fig 2:**
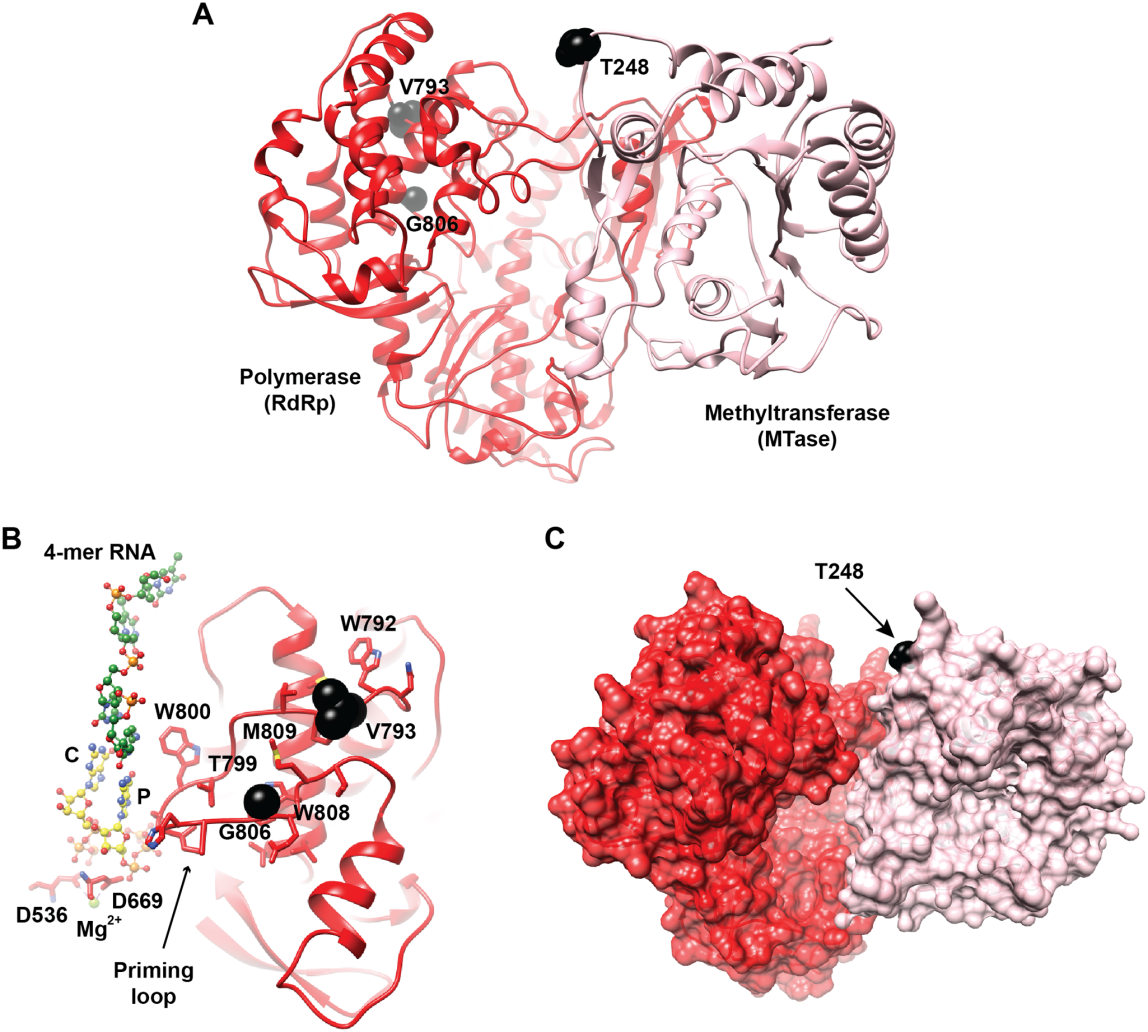
Location of the mutation sites associated with altered fidelity in WNV NS5. **A.** Shown are the crystal structures of the N-terminal methyltransferase domain (MTase, colored pink; PDB 2OY0) and the C-terminal polymerase domain (RdRp, colored red; PDB 2HFZ) of the NS5 protein from WNV. The relative positioning of the two domains was based on the crystal structure of the full length NS5 protein of DENV (PDB 4V0R). The protein is represented as ribbon and the identified mutants (T248, V793, and G806) are represented as black spheres. The amino acids V793 and G806 are located in the priming loop that is part of the thumb subdomain. **B.** The initiation model of WNV RdRp showing the thumb subdomain of the polymerase, rotated 180° compared to the view in A, a 4-mer RNA extracted from the ɸ6-RdRp (PDB 1HI0, green carbon atoms), rNTP modeled at the priming site (P) and the catalytic site (C) based on the complex structure of HCV RdRp (PDB G1X5, yellow carbon atoms), and the active-site aspartates D636 and D669 with the bound catalytic Mg^2+^ ion. The substitutions, V793I and G806R, are expected to impact the interactions of the nearby residues of the priming loop, including W792, T799, W808, and M809. Disruption of these interactions would affect the dynamics of the priming loop, including the conformational change of W800 that is suggested to be required for stabilizing the initiation complex. **C.** the MTase and polymerase domains shown in (A) are rendered as pink and red surfaces, respectively, to emphasize the expected large interface between the two domains. Residue T248 is colored black; substitution of this residue by an isoleucine could impact the interface between the two domains of NS5 structure. The figure was prepared using program CHIMERA.

In order to identify shared WNV amino acid (aa) substitutions associated with mutagen resistance, full-genome sequencing of clonal strains WNV pp3 and WNV pp9 was completed. A total of 15 (pp3) and 18 (pp9) nt substitutions were identified, resulting in 9 and 8 aa substitutions, respectively (Table 2). Of these, 10 nt and 7 aa substitutions were shared. Given the assumption that substitutions outside of the replication complex were more likely to be associated with adaptation to Hela cell culture or drift, shared aa substitution in the WNV RdRp and methyltransferase (Mtase) genes exclusively were chosen for further characterization. These included C8423T, resulting in a threonine to isoleucine change at position 248 of the Mtase, as well as G10057A and G10096A, resulting in valine to isoleucine and glycine to arginine changes at positions 793 and 806 of the RdRp, respectively (Table 2). Mapping of these residues on the known flavivirus RdRp and Mtase structures demonstrates that T248I is located at the C-terminal loop (aa 245–267), which is expected to interact with the RdRp domain, and both V793I and G806R exist in locations outside of the RdRp active site, although are within the priming loop (Fig 2; [39–42]). Both T248 and G806 are conserved among lineage I WNV strains, yet not across lineages or species, while V793 is shared among flaviviruses. No naturally circulating strains were found to possess the identified mutations at these locations.

**Table 2:** Mutations identified in clonal WNV populations with decreased ribavirin susceptibility. Nucleotide (NT) and amino acid (AA) mutations identified in biological clones with decreased ribavirin susceptibility. Shared AA substitutions identified in the WNV replication complex, including RNA-dependent-RNA-polymerase (RdRp) and methyltransferase (Mtase) changes, are highlighted.

WNV pp3			WNV pp9	
NT change	AA change	Gene	NT change	AA change
G1459A	G-R	ENV	G1459A	G-R
G1522A		ENV	G1522A	
C1569T		ENV		
T1724C	I-T	ENV		
G1783A	E-K	ENV	G1783A	E-K
		ENV	C2079T	
		ENV	C2323T	
C2370T		ENV		
G2721A		NS1	G2721A	
C4388T	T-M	NS2B	C4388T	T-M
A4655G	K-R	NS3	A4655G	K-R
		NS3	C5798T	
		NS3	C5823T	
		NS3	C6120T	
		NS4A	G6721A	A-T
C7069T	L-F	NS4B		
		NS4B	C7275T	
***C8423T***	***T248I***	***NS5 (Mtase)***	***C8423T***	***T248I***
G8997T		NS5 (RdRp)		
		NS5 (RdRp)	C9660T	
***G10057A***	***V793I***	***NS5 (RdRp)***	***G10057A***	***V793I***
***G10096A***	***G806R***	***NS5 (RdRp)***	***G10096A***	***G806R***
C10529T		3' UTR	C10529T	

**Fig 3:**
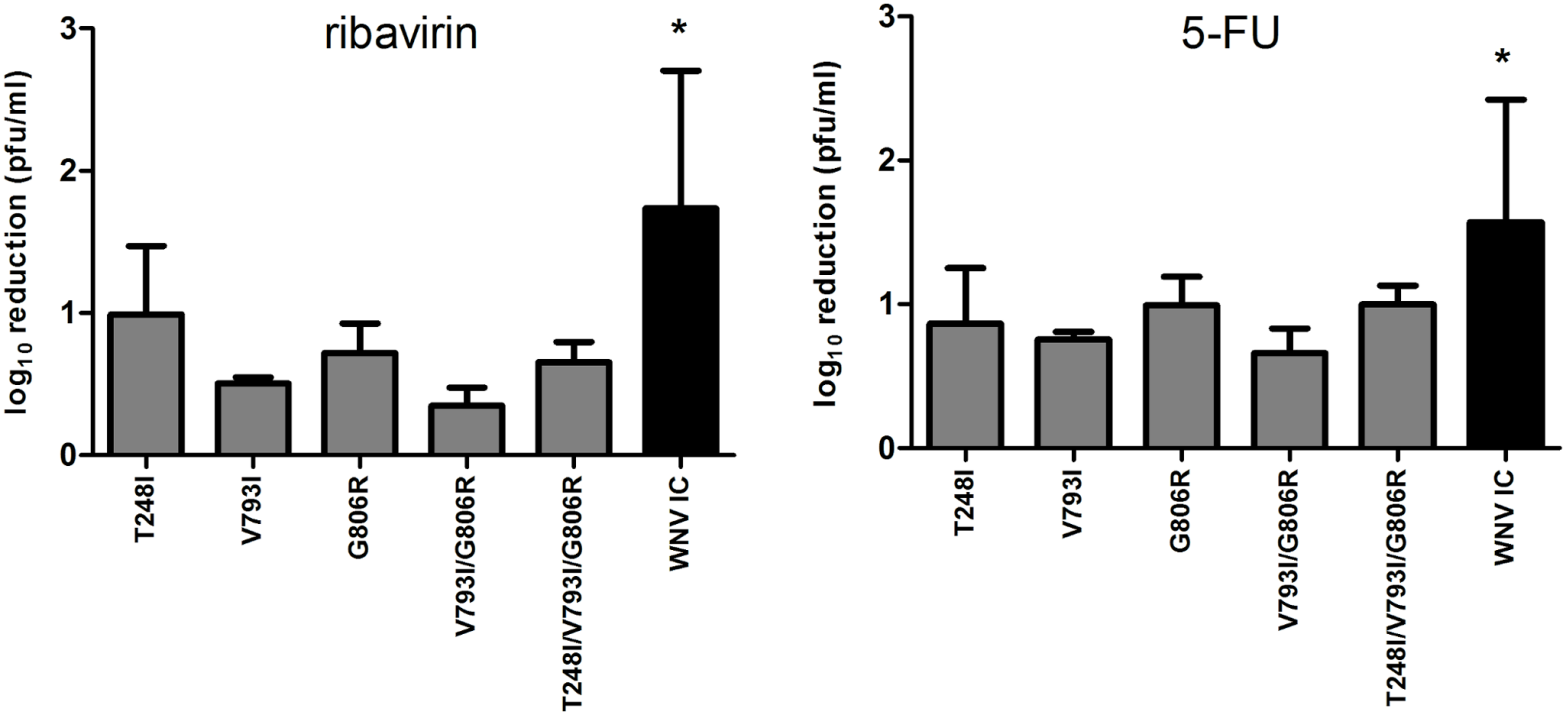
Decreased mutagen susceptibility of WNV replication complex mutants. RdRp and Mtase mutations identified in WNV populations with the highest ribavirin resistance were reverse engineered into the WNV-IC individually (T248I, V793I and G806R) and in combination (V793I/G806R, T248I/V793I/G806R) and assessed for mutagen resistance on Hela cell culture using either 100uM ribavirin or 50uM 5-fluorouracil (5-FU). Bars depict mean reduction in titer +/- standard deviation following mutagen treatment. Significantly higher viral titers (*t-test, df = 5, p<0.05) were measured for WNV-IC relative to all WNV mutants after 72h treatment with both antivirals.

To confirm that these amino acid substitutions independently conferred decreased ribavirin susceptibility, and to assess if this corresponded to generalized mutagen resistance, mutations were engineered independently and in combination into the WNV-IC and susceptibility to both ribavirin and 5-fluorouracil was assessed with WNV mutants. Full-genome sequencing following mutagenesis confirmed the exclusive presence of desired mutations. WNV mutants created included WNV T248I, V793I, G806R, double mutant V793I/G806R, and triple mutant (T248I/V793I/G806R). Mutagen resistance assays demonstrated significantly decreased reduction in titer (susceptibility) relative to untreated controls for all mutant strains as compared to WNV-IC following treatment with both ribavirin and 5-fluorouracil (t-test, df = 6 p<0.05; Fig 3). The highest level of mutagen resistance was measured with the RdRp double mutant, WNV V793I/G806R, for which 2.2 and 4.6-fold mean titer reduction were measured following ribavirin and 5-fluorouracil treatments, respectively, as compared to 55 and 37-fold mean titer reductions measured with WNV-IC (Fig 3).

### Mutant swarm characterization

**Fig 4:**
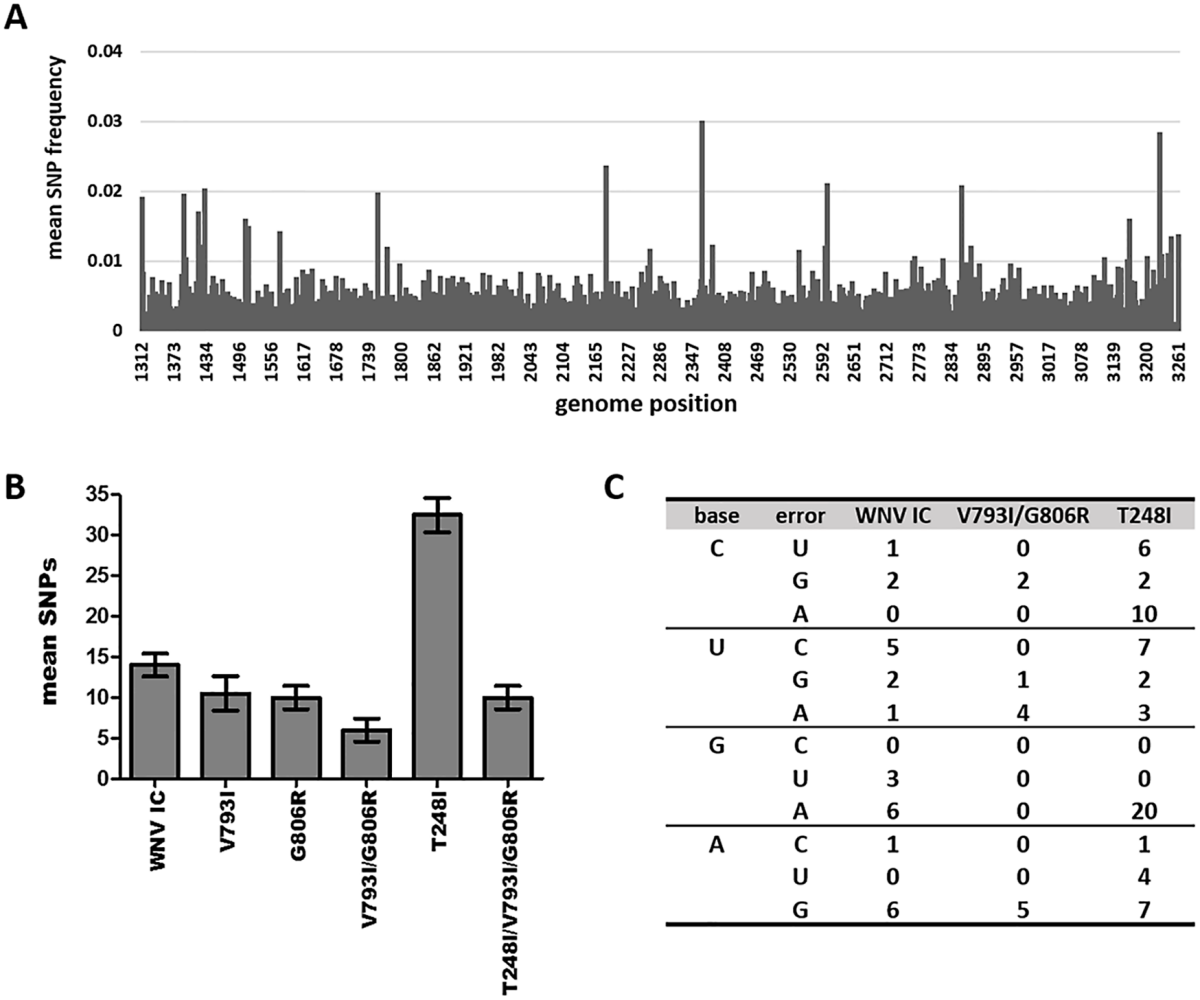
Strain-specific differences in mutational spectra breadth and composition. WNV mutant swarm characterization by deep-sequencing of nucleotides 1312–3261 (ENV/NS1) following 72h growth on mosquito cell culture. **A.** Mean distribution of genomic diversity (single nucleotide polymorphisms [SNP]) of 2 replicates of WNV-IC across the sequenced region. The distribution of mutations was similar among strains. **B.** Mean number of minority SNPs identified for individual WNV strains at frequencies greater than 0.5%. **C.** Distribution of substitution types among WNV strains.

In order to test the hypothesis that mutagen resistance corresponded to alterations in mutation rate, deep-sequencing was used to quantify accumulation of unique SNPs following a single passage on mosquito cell culture (Fig 4A). Levels of WNV RNA for both WNV-IC and mutant strains were statistically similar for all samples chosen for sequencing (~9.0 log_10_ WNV copies/ml), suggesting differences in replication were not likely to account for differences in the number of mutations accumulated. Assays were completed in duplicate for each WNV strain and sequencing results identified fewer SNPs in all mutant strains relative to WNV-IC, with the exception of the methyltransferase mutant, WNV T248I, for which the number of unique SNPs identified was approximately 2.5 fold higher than WNV-IC. The RdRp double mutant, WNV V793I/G806R, showed the fewest number of unique SNPs; approximately 2.5 fold lower than WNV-IC (Fig 4B). Mutations were distributed throughout the sequenced regions for all WNV strains, yet mutant swarm composition varied significantly among strains (Fig 4C). Specifically, transition to transversion ratios were ~2:1 for WNV-IC and WNV T248I, yet <1 for WNV V793I/G806R. Decreased mutation of WNV V793I/G806R relative to WNV-IC resulted primarily from lack of U to C and G to A mutations, which accounted for 11/28 mutations for WNV-IC and 0/12 mutations for WNV V793I/G806R (chi-squared, p = 0.011). Increased mutation of WNV T248I, on the other hand, resulted primarily from A misincorporations, which accounted for 33/62 mutations for WNV T248I, and just 7/28 mutations of WNV-IC (chi-squared, p = 0.045). Approximately the same number of U to A mutations were identified for WNV V793I/G806R as WNV T248I (4 vs 3), yet no G to A mutations were identified for WNV V793I/G806R, in stark contrast to the 20 identified for WNV T248I (Fig 4C). These results demonstrate strain-specific differences in the misincorporation of different nucleotides and, more specifically, in the propensity for particular mispairs, suggesting mutation frequencies may be dependent on both replication complex and template sequences and/or context.

### Biochemical characterization

**Fig 5:**
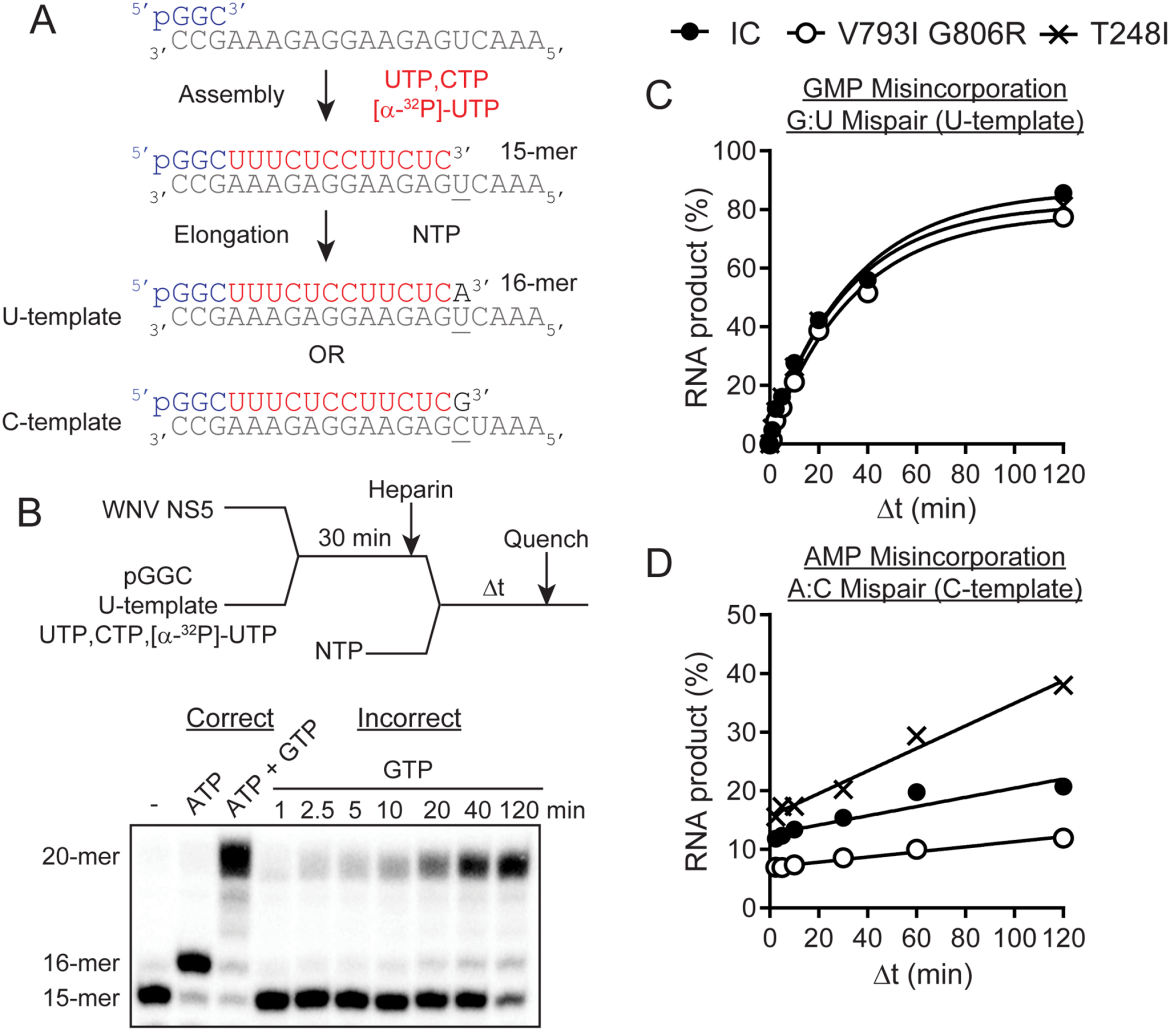
Evaluation of WNV NS5 variants with an in vitro primer extension assay reveals differences in polymerase incorporation fidelity. **A.** Schematic of primer extension assay for evaluating WNV NS5 polymerase activity. **B.** Evaluation of elongation reactions using either correct or incorrect nucleotide substrates. Shown is the experimental design and denaturing PAGE gel of both correct and incorrect nucleotide addition. Experimental design: WNV NS5 is assembled for 30 min to produce elongation-competent complexes at which point a trap (heparin) for free enzyme is added. The reaction is then rapidly mixed with NTP substrate and quenched at various times. Denaturing PAGE gel: The 15-mer RNA is rapidly extended to 16-mer RNA product in the presence of correct nucleotide substrate, ATP. The presence of both ATP and GTP together promote full extension to 20-mer RNA as the terminal templating bases are competent for correct nucleotide addition. The presence of GTP alone allows for the kinetics of incorrect nucleotide incorporation to be observed (G:U mispair). **C.** Comparison of the kinetics of misincorporation between WNV-IC, V793I/G806R and T248I NS5. There is no observable difference in the kinetics of GMP misincorporation (G:U mispair). Shown is the percentage of RNA product plotted as a function of time and fit to a single exponential. **D.** By using an alternative substrate that allows for A:C mispairs to be evaluated, a difference in the efficiency of AMP misincorporation is observed between the three NS5 proteins.

Purified IC, V793I/G806R, and T248I WNV NS5 proteins were used to quantify and compare the kinetics of nucleotide misincorporation of NS5 elongation—competent complexes (Fig 5). Complexes were assembled using a 5’-phosphorylated trinucleotide primer (pGGC), single stranded RNA template, UTP and CTP (Fig 5A). The template was designed such that two nucleotides led to production of a 15-mer RNA. Labeling of the elongation complex was achieved by using α-^32^P-labeled nucleotide. Once assembled, the complex was stable and capable of rapid incorporation of the next correct nucleotide substrate (elongation) to produce a 16-mer RNA product. The elongation competent complex was then used to interrogate the kinetics of nucleotide misincorporation. The initial substrate, designed to measure G:U mispairs was used to quantify GMP misincorporation for each NS5 protein (Fig 5B and 5C). Comparing the percentage of RNA product produced over time, it was demonstrated that WNV-IC, V793I/G806R and T248I NS5 proteins displayed similar *in vitro* kinetics for GMP misincorporation (Fig 5C). These results were consistent with a lack of biological differences in fidelity among NS5 variants, but were also in agreement with deep-sequencing data, for which the number of A to G substitutions were similar among WNV-IC, WNV V793I/G806R, and WNV T248I (Fig 4C). In order to evaluate fidelity differences implied by deep-sequencing data, the template for biochemical assays was redesigned to quantify A:C mispairs, equivalent to G to A substitutions. The number and proportion of G to A substitutions identified following growth in mosquito cells differed among strains, with means of 6, 0 and 20 identified in WNV-IC, WNV V793I/G806R, and WNV T248I, respectively (Fig 4C). Biochemical assays were consistent with these results, clearly demonstrating an increasing rate of A misincorporation for WNV T248I relative to WNV-IC and a decreasing rate of A misincorporation for WNV V793I/G806R (Fig 5C). Taken together, these results demonstrate sequence-specific fidelity differences among WNV mutant strains.

### *In vitro* growth kinetics

**Fig 6:**
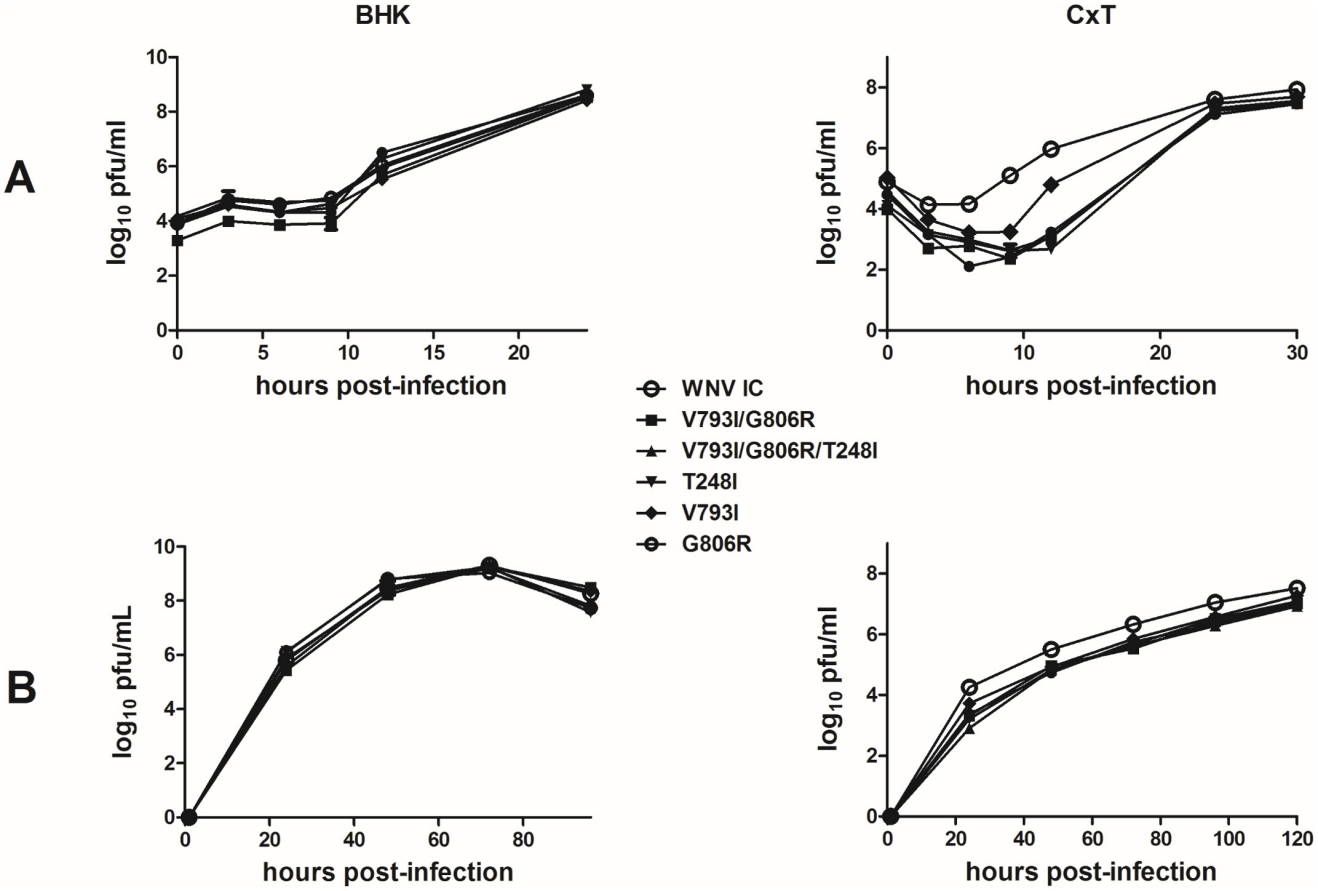
Host-specific effects of WNV replication complex mutations on replicative fitness *in vitro*. Growth kinetics of WNV strains in vertebrate (BHK) and mosquito (CxT) cell culture are shown for both 1-step (**A**; MOI 10 and 8, respectively) and multi-step (**B**; MOI 0.01) infections. Viral growth kinetics were equivalent in BHK cells and significantly different in CxT cells (repeated measures ANOVA, p<0.001). Specifically, WNV-IC titers were significantly higher in CxT cells at both MOIs relative to all mutant strains (ANOVA, Tukey post-test, p<0.05).

**Fig 7:**
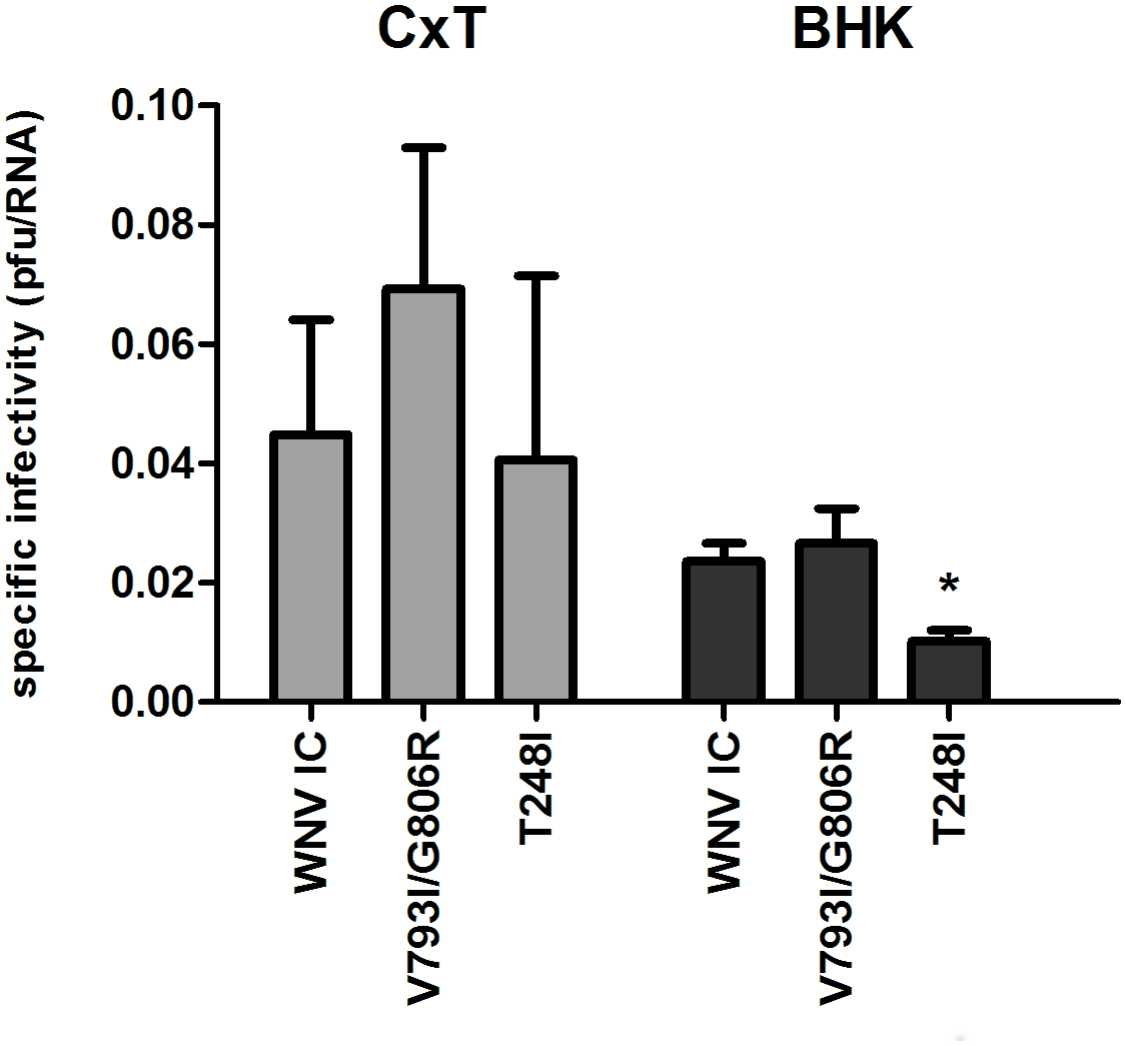
Specific infectivity of WNV replication complex mutants. WNV RNA (viral particles) and infectious virus (pfu) were quantified following 1-step growth on mosquito (CxT) or vertebrate (BHK) cell culture and specific infectivity (particle/pfu) was compared among WNV-IC, WNV V793I/G806R and WNV T248I. Graphs represent means +/- standard deviation. Significantly decreased infectivity (*) was measured for WNV T248I relative to WNV-IC and WNV V793I/G806R (t-test, df = 4, p = 0.0028).

Comparison of one-step and multi-step growth kinetics of WNV mutants to WNV-IC in vertebrate (BHK) and invertebrate (CxT) cell lines demonstrates host-specific effects of replication complex mutations (Fig 6). No differences in overall kinetics (repeated measures ANOVA, F = 0.14, df = 6, p = 0.98) or viral titers at individual time points (t-test, p>0.05) were measured when comparing WNV mutants to WNV-IC on vertebrate cell culture, while significant differences in viral kinetics were measured in mosquito cell culture at both MOIs for all replication complex mutants relative to WNV-IC (repeated measures ANOVA, F = 12.83, df = 6, p<0.0001). Specifically, consistently lower viral titers were measured for all RdRp and Mtase mutants in mosquito cells relative to WNV-IC (Tukey’s multiple comparison test, p<0.05), and kinetics were similar among mutants with the exception of WNV V793I, which despite having significantly lower titers than WNV-IC had modestly higher titers relative to other mutants (Fig 6). In addition, WNV RNA was quantified with qRT-PCR following one-step growth and particle/pfu ratios were quantified and compared among WNV-IC, WNV V793I/G806R and WNV T248I in order to assess the relationship between fidelity and infectivity. Specific infectivity was elevated in mosquito cells as compared to vertebrate cells for all WNV strains (Fig 7). Trends measured with infectivity are consistent with identified fidelity differences in both cell lines, with the highest infectivity measured with the RdRp double mutant WNV V793I/G806R and the lowest infectivity measured with the Mtase mutant WNV T248I, yet differences were only significant relative to WNV-IC for WNV T248I in BHK cells (t-test, df = 4, p = 0.0028; Fig 7). These differences do not therefore account for differences in growth kinetics identified among WNV strains in CxT cells.

### Vector competence

In order to determine if modest attenuation in mosquito cell culture and fidelity differences corresponded to differences in mosquito competence *in vivo*, infectivity of WNV-IC was compared to infectivity of WNV T248I and WNV V793I/G806R in colonized *Cx*. *quinquefasciatus* mosquitoes following exposure to infectious bloodmeals. Input titers were comparable to a natural dose and similar among WNV strains and experimental replicates (Table 3; [43]). Despite the fact that levels of infection were somewhat lower than have been measured with other wildtype WNV strains (35.8%), stark and highly significant differences were measured when compared to WNV mutants (chi-squared, p<0.001 for all mutants relative to WNV-IC). Specifically, over the course of 3 experimental replicates and multiple time points, a total of just 2 of 210 (V793I/G806R) and 3 of 203 (T248I) mosquitoes acquired measurable infections. To confirm that this was generalizable phenotype which was relevant in natural populations, infectivity experiments were repeated in *Cx*. *quinquefasciatus* recently acquired from the field. Although modestly lower input titers were used, infectivity was slightly higher for WNV-IC relative to colonized mosquitoes (39.2%) and a general lack of infectivity was confirmed for replication complex mutants (Table 3). Given the inefficient infectivity of mutant strains, dissemination and transmission were not evaluated in this study.

**Table 3:** WNV infection rates (total WNV positive/total tested) of *Cx*. *quinqufasciatus* following exposure to an infectious bloodmeal.

population	WNV strain	BM titer (log10/ml)	Day 4 or 5 (%)	Day 7 (%)	Day 10 (%)	Day 12 or 14 (%)	TOTAL (%)
**colony**	WNV-IC	*7*.*8*	*30/110 (27*.*3)*	*30/100 (30)*	*31/69 (44*.*9)*	*37/79 (46*.*8)*	*128/358* (35.8)
	V793I/G806R	*7*.*7*	0/70 (0)*	1/60 (1.7)*	1/40 (2.5)*	0/40 (0)*	2/210 (1.0)*
	T248I	*7*.*5*	1/40 (2.5)*	0/52 (0)*	1/59 (1.7)*	2/52 (3.9)*	3/203 (1.5)*
**field**	WNV-IC	*7*.*4*	*nc*	*11/35 (31*.*4)*	*nc*	*18/39 (46*.*2)*	*29/75* (39.2)
	V793I/G806R	*7*.*4*	*nc*	1/35 (2.9)*	*nc*	0/40 (0)*	1/75 (1.3)*
	T248I	*7*.*3*	*nc*	1/36 (2.8)*	*nc*	1/42 (2.4)*	2/78 (2.6)*

nc- not completed

*Chi-squared, p<0.0001

Although viral load was not determined for individual mosquitoes, it is notable that the 8 total mosquitoes identified as positive following exposure to WNV mutants all showed relatively low levels (less than 20 plaques) with undiluted plaque screens.

## Discussion

As has been successfully accomplished with other systems [21,22,24,25,44], we exploited selection in the presence of a mutagen to identify mutations altering WNV replicase fidelity and utilized WNV fidelity mutants to interrogate the consequences and mechanisms of altered mutation rates. Although ribavirin is not considered an efficacious antiviral for the treatment of active WNV infections, it has been shown to cause both error-prone replication and WNV attenuation *in vitro*, particularly in Hela cell culture [45].

The relative decrease in mutation frequency measured for the WNV high fidelity variant V793I/G806R (~2.5 fold) is similar or modestly higher than has been shown with other systems [21,22,24,25,44]. With the exception of coronaviruses, which employ a proofreading exoribonuclease system unique among RNA viruses [46], these data together demonstrate that either the lack of biochemical capacity or the extent of phenotypic consequences by-in-large prevent highly significant alterations to RdRp fidelity. Despite this, data presented here and in previous studies clearly demonstrate that subtle alterations to mutation rates can have profound phenotypic effects on RNA viruses.

Although fully characterizing the mechanism by which these RdRp residues alter fidelity would require further biochemical and biophysical investigations, mapping of T248, V793 and G805 residues on the crystal structures of NS5 [41,42] provides some indication of possible mechanisms (see Fig 2). Despite the lack of full-length NS5 structure from WNV, the relative orientation of the Mtase domain with respect to the polymerase domain can be defined using the recent crystal structure of the full length dengue virus (DENV) NS5 [47] as a guide. The two NS5 structures are highly similar with an RMSD of 1.18 and 0.65 Å for the polymerase and Mtase domains, respectively. The residues V793 and G805 are located in the priming loop (aa 789–812), which is a long loop that links two α-helices in the thumb subdomain and protrudes to reach the active site. Conformational dynamics of the priming loop is believed to be necessary to form a stable initiation complex [42]. A model of the initiation complex is shown in Fig 2B; in this model a 4-mer ssRNA substrate, taken from the complex structure of the related ɸ6-RdRp (PDB 1HI0) [48], and rNTP modeled at the priming site (P-site) and catalytic site (C-site) based on the HCV RdRp complex structure (PDB 1GX5) [40] can be accommodated in the WNV NS5 RdRp active site with minimal steric clashes with the protein atoms. To form a stable initiation complex, the active-site residue Trp-800 would alter its sidechain conformation to be able to stack against the priming nucleotide. The priming loop maintains numerous interactions with residues from the thumb, fingers and palm subdomains and substitutions similar to V793I and G806R could potentially disrupt these interactions, impacting the dynamics of the priming loop and subsequently affecting the initiation process of the RdRp. It is not difficult to conceive that V793I and G806R may restrict the dynamics of the priming loop, leading to a higher fidelity mutant in a scenario similar to what has recently been shown for the G64S high-fidelity mutant of PV RdRp [49,50]. In these studies of PV RdRp, remote site mutations altered the polymerase fidelity by causing changes to the dynamics of conserved structural elements and motifs including residues at the active site. Although the interactions of the flavivirus RdRp and Mtase have now been well-documented [51,52] the finding that modifications to allosteric interactions resulting from mutation of a single residue of the Mtase can significantly alter replication fidelity is novel. The T248I mutation is located at the C-terminal loop (aa 245–267) of the MTase domain; which is expected to be at the interface between the two domains of the WNV NS5 (Fig 2C), similar to what is observed in the homologous DENV NS5 structure. The loop harboring T248 is predicted to interact with the region of the fingers in the polymerase domain (aa 350–365). It is very likely that amino acid substitution of T248 by an isoleucine could affect the interactions between the two domains and the inter-domain dynamics, eventually affecting the polymerase active site and altering fidelity. Findings with WNV are therefore consistent with previous data demonstrating that RdRp fidelity is determined by a complex network of interactions and checkpoints by which remote site mutations may alter the dynamics of conserved structural elements and motifs including residues at the active site [9,49,53,54].

Although selection for ribavirin resistance did, as predicted, result in the isolation of high fidelity WNV variants, the fact that a mutator variant also displayed resistance could be explained by antiviral mechanisms independent of lethal mutagenesis for WNV in this system. Similar results were attained with FMDV, for which a low fidelity RdRp was found to have a decreased capacity for ribavirin incorporation [55]. In addition, previous studies with another flavivirus, yellow fever virus, demonstrate that the antiviral actions of ribavirin are conferred primarily by the depletion of intracellular GTP pools [56]. Additional antiviral mechanisms of ribavirin have also been proposed, including inhibition of virus transcription [57] and inhibition of both guanyltranferase and Mtase activity [58,59]. The flavivirus Mtase is required for RNA capping [60], a process partially enabled by GTP binding [61] and competitively inhibited by ribavirin with DENV NS5 [58]. Although T248 is not within the nucleotide binding site it is possible that this mutation could perturb these interactions and subsequently interfere with antiviral susceptibility in this manner. On the other hand, given that WNV T248I also displays resistance to 5-fluorouracil, it is possible that the strain-dependent mutational biases could result in unique evolutionary trajectories and, subsequently, strain-specific differences in mutational robustness and susceptibility to lethal mutagenesis.

This sequence-dependent nature of the fidelity alterations also demonstrates that broad assumptions about fidelity and mutagen susceptibility likely discount the specificity of interactions of individual nucleotides and/or base analogs with the replication complex. Although others have demonstrated that modifications to fidelity are attainable, the possibility that unique strains may possess unique mutational biases has novel functional and evolutionary implications. Specifically, if mutational landscapes are strain-specific, so too are fitness landscapes of viral swarms and therefore evolutionary pressures acting on them. Such biases could be exploited by evolution as a means of increasing the probability of producing favorable mutant swarms following genetic bottlenecks or could have the opposite effect of constraining deleterious strains by not permitting adequate exploration of sequence space to escape unfit landscapes.

Consistent with previous studies with CHIKV [24] *in vitro* kinetics were generally similar for the high fidelity WNV V793I/G806R relative to WNV-IC, with modest attenuation measured in mosquito but not vertebrate cell culture. Although fitness differences were only measurable with direct competition of CHIKV and not individual growth assays, the decreases in mutation rate measured for WNV V793I/G806R were also more substantial than those measured for CHIKV, likely due to combining two RdRp mutations which appear to have an additive effect on fidelity. These host-specific effects are consistent with previous studies demonstrating increased swarm diversity in the mosquito for both WNV and its close relative St. Louis encephalitis virus [31,62], but further suggest that the invertebrate environment is not simply a more robust environment which tolerates diversity, but one in which diversity itself likely provides a fitness benefit [30]. It is possible that this fitness benefit results from an inherent need to escape RNAi or other innate invertebrate immune responses [63], or that enhancements in fitness could result from cooperative interactions among distinct genotypes and viral proteins [64]. Despite this, previous passage studies in *Cx*. *pipiens* suggest that this need for diversity may be overcome by individual variants with highly superior fitness [28] and results presented here demonstrating attenuation of the low fidelity mutant WNV T248I suggest, not surprisingly, that there is a limit to the benefit of diversity. The association of mutator phenotypes with either similar or attenuated viral growth kinetics is consistent with what has been observed in other systems [17–19], and gives further credence to the idea that replication and mutation rate are not necessarily inextricably bound phenotypes. Given that vertebrate environments have been found to be more restrictive both *in vitro* and *in vivo* [32,65], it is somewhat surprising that a virus with a mutator phenotype would not also be attenuated in vertebrate cell culture, yet even if WNV T248I is more mutationally robust than WNV-IC, attenuation may be observed if this strain were repeatedly passaged, therefore accumulating diversity and, presumably, deleterious mutants [17,18]. Consistent with this is the fact that the Mtase mutant was also found to be less infectious in vertebrate cell culture. In addition, competition assays with increased sensitivity for detecting more subtle fitness differences [24,66] or *in vivo* models that more accurately represent natural infections could reveal important phenotypic differences in vertebrate systems [67]. Although in the current studies results confirm that inherent biochemical differences account for differences in mutation rate independent of cell type, it is also feasible that fidelity itself could be host-dependent, as a recent study with vesicular stomatitis virus demonstrates slower mutation rates in insect cells as compared to mammalian cells [27]. Although few have investigated this concept [68], it is not necessarily surprising that the biophysical and biochemical properties of the replication complex might differ significantly in environments with variable temperature, pH, and nucleotide availability. Future studies exploiting new sequencing technologies to evaluate mutation rates in a range of systems will help to clarify these differences [17,69,70].

Although the modest attenuation in mosquito cell culture may be explained by the modest alterations to fidelity, it is much more surprising that an approximately 2.5 fold alteration to mutation rate could almost entirely eliminate the capacity for infection and/or sustainable WNV replication in mosquitoes. Although studies with CHIKV also demonstrate that fidelity variants are associated with decreased infectivity in mosquitoes, differences measured for WNV here are much more profound. These results suggest either that WNV replication in gut epithelial cells is uniquely sensitive to alterations in fidelity or that alternative mechanisms of attenuation related to host interaction with the flavivirus NS5 exist. Regardless, these variants provide powerful tools to elucidate the determinants of flavivirus mosquito competence and novel targets for viral attenuation.
